# Prevalence and characteristics of *pks* genotoxin gene cluster-positive clinical *Klebsiella pneumoniae* isolates in Taiwan

**DOI:** 10.1038/srep43120

**Published:** 2017-02-24

**Authors:** Ying-Tsong Chen, Yi-Chyi Lai, Mei-Chen Tan, Li-Yun Hsieh, Jann-Tay Wang, Yih-Ru Shiau, Hui-Ying Wang, Ann-Chi Lin, Jui-Fen Lai, I-Wen Huang, Tsai-Ling Lauderdale

**Affiliations:** 1 Institute of Genomics and Bioinformatics, National Chung Hsing University, Taichung, Taiwan; 2 Institute of Molecular and Genomic Medicine, National Health Research Institutes, Zhunan, Taiwan; 3 Biotechnology Center, National Chung Hsing University, Taichung, Taiwan; 4 Department of Microbiology and Immunology, Chung-Shan Medical University, Taichung, Taiwan; 5 Division of Infectious Diseases, Department of Internal Medicine, Chung-Shan Medical University Hospital, Taichung, Taiwan; 6 Institute of Microbiology and Immunology, Chung-Shan Medical University, Taichung, Taiwan; 7 National Institute of Infectious Diseases and Vaccinology, National Health Research Institutes, Zhunan, 350 Taiwan; 8 Department of Internal Medicine, National Taiwan University Hospital, Taipei, Taiwan

## Abstract

The *pks* gene cluster encodes enzymes responsible for the synthesis of colibactin, a genotoxin that has been shown to induce DNA damage and contribute to increased virulence. The present study investigated the prevalence of *pks* in clinical *K. pneumoniae* isolates from a national surveillance program in Taiwan, and identified microbiological and molecular factors associated with *pks*-carriage. The *pks* gene cluster was detected in 67 (16.7%) of 400 isolates from various specimen types. Multivariate analysis revealed that isolates of K1, K2, K20, and K62 capsular types (*p* < 0.001), and those more susceptible to antimicrobial agents (*p* = 0.001) were independent factors strongly associated with *pks*-carriage. Phylogenetic studies on the sequence type (ST) and pulsed-field gel electrophoresis patterns indicated that the *pks*-positive isolates belong to a clonal group of ST23 in K1, a locally expanding ST65 clone in K2, a ST268-related K20 group, and a highly clonal ST36:K62 group. Carriage of *rmpA, iutC*, and *ybtA*, the genes associated with hypervirulence, was significantly higher in the *pks*-positive isolates than the *pks*-negative isolates (95.5% vs. 13.2%, *p* < 0.001). Further studies to determine the presence of hypervirulent *pks*-bearing bacterial populations in the flora of community residents and their association with different disease entities may be warranted.

*Klebsiella pneumoniae* is not only a major nosocomial pathogen but also an important cause of community-acquired infections^1^,^2^,^3^. Some geographic differences in disease caused by *K. pneumoniae* infection exist. One example is *Klebsiella* liver abscess (KLA), an invasive disease with severe complications^3^,^4^. KLA is more prevalent in Asia, particularly in Taiwan^3^,^4^,^5^,^6^,^7^, but is less common in other continents^7^,^8^,^9^. The differences in *K. pneumoniae* disease prevalence have been associated with the ethnic background or genetic predisposition of the host and differences in virulence potentials of the organism types in each geographic region^3^,^10^.

Among the 79 distinct capsular types of *K. pneumoniae*^11^, the hypervirulent strain of K1 is highly associated with KLA, and K2 is the second most commonly associated type^12^,^13^. In contrast, K1 and K2 *K. pneumoniae* strains are still relatively rare in other parts of the world^14^,^15^. A few other capsular types, K5, K20, K54 and K57, have also been associated with KLA invasive diseases^16^. These virulent types (K1, K2, K5, K20, K54, and K57) are also prevalent in community-onset *K. pneumoniae* pneumonia^17^. The presence of *rmpA, iutC, ybtA*, and *pks,* the genes responsible for hypermucoviscosity, production of iron-acquisition factors aerobactin and yersiniabactin, and production of colibactin genotoxin, respectively, have been associated with hypervirulence in *K. pneumoniae*^7^,^18^,^19^,^20^,^21^,^22^.

Colibactin, a hybrid peptide-polyketide genotoxin, was first identified in extraintestinal pathogenic *E. coli* strain IHE3034^23^. Subsequently, it was shown that colibactin could induce double strand DNA breaks in lymphocytes to result in cell cycle arrest and cell death^24^. The genes responsible for colibactin synthesis were found on a gene cluster, referred to as *pks* colibactin gene cluster, which is located at the *asnW* tRNA locus in the *E. coli* genome^23^. According to a study in Europe, the *pks* colibactin gene cluster was detected in 34% of *E. coli* strains of phylogenic lineage B2, but only in 3.5% of *K. pneumoniae* clinical isolates^25^. However, data on the prevalence of the *pks* gene cluster in different capsular types of *K. pneumoniae* from different sources are limited.

Recently, sequencing of *K. pneumoniae* strain 1084 from Taiwan revealed the presence of a *pks* gene cluster that is identical to the *E. coli* IHE3034 version^26^. The *pks* gene cluster was found in a 208-kb high pathogenicity island (HPI), named pKPHPI208, at the *asn* tRNA loci of the *K. pneumoniae* 1084 chromosome (GenBank Accession No. NC_018522.1). The KPHPI208-like genomic island has been linked to the emerging hypervirulent *K. pneumoniae* lineage and may also be involved in increased virulence^27^. Using the *clbA* mutants constructed from *K. pneumoniae* 1084, Lai *et al*., showed that the *pks* colibactin genes were involved in DNA damage in the host^28^. In the same study, a high prevalence (25.6%) of the *pks* colibactin gene cluster was found among *K. pneumoniae* clinical isolates, a rate significantly higher than the 3.5% found in a recent European survey^25^. However, the isolates in the study by Lai *et al*. were from a single hospital in Taiwan in 2002, thus may not represent the current national trend. The present study was carried out to investigate the prevalence of the *pks* colibactin gene cluster in *K. pneumoniae* clinical isolates from a multicenter national surveillance program^29^. Microbiological and molecular characterizations were also performed to identify factors associated with *pks* gene cluster carriage.

## Results

### Prevalence of *pks* gene cluster and capsular types among *K. pneumoniae* clinical strains

A total of 400 *K. pneumoniae* clinical isolates comprising 100 each of blood, respiratory, urine, and other specimen sources from 2012 were randomly selected for this study. Among them, 67 (16.8%) isolates were *pks*-positive including 17, 26, 9, and 15 isolates from blood, respiratory, urine, and other specimen groups, respectively. The capsular type distribution and *pks* positivity stratified by specimen origin are listed in Table 1. Among these 400 isolates, 33 (8.3%) were K1 and 45 (11.3%) were K2, 12 (3.0%) were K5, 23 (5.8%) were K20, 20 (5.0%) were K54, 8 (2.0%) were K57, and 25 (6.3%) were K62. The *pks* gene cluster was detected in 26 (78.8%), 19 (42.2%), 11 (47.8%), 1 (12.5%), and 9 (36.0%) of the K1, K2, K20, K57, K62 capsular type isolates, respectively. The capsular type of the remaining 1 *pks*-positive isolate could not be identified and the capsular types of the 234 non-K1, K2, K5, K20, K57, K62 *pks*-negative isolates were not determined.

### Cocarriage of *pks* and hypervirulence genes

To test for co-carriage of *pks* and hypervirulence factors, we performed PCR to determine the presence of *iucA* (aerobactin), *rmpA* (hypermucositity), and *ybtA* (yersiniabactin), the genes associated with hypervirulence, Among the 67 *pks*-positive isolates, 64 (95.5%) were positive for *rmpA, iutC*, and *ybtA*, The remaining 3 isolates (all K2) were *ybtA*-positive and *iutC*-negative, and 2 of which were also *rmpA*-negative. In the 333 *pks*-negative isolates, only 13.2% (44) were *iutC*/*rmpA*/*ybtA*-positive. Thus the *pks*-positive isolates had significantly higher carriage rate of *rmpA, iutC*, and *ybtA*, than the *pks*-negative isolates (95.5% vs. 13.2%, *p* < 0.001).

### Antimicrobial susceptibility of *pks*-positive and *pks*-negative isolates

The *pks*-positive *K. pneumoniae* isolates were significantly more susceptible to 13 of the 14 antimicrobial agents tested (Table 2), including aminoglycosides, β-lactams, β-lactam/β-lactamase inhibitors, fluoroquinolones, and folate pathway inhibitors. For example, susceptibility to cefazolin, ciprofloxacin, gentamicin, and trimethoprim/sulfamethoxazole was 94.0%, 100%, 100%, and 98.5% vs. 66.4%, 69.7%, 71.8%, and 64%, respectively, in the *pks*-positive vs. *pks*-negative isolates (all *P* < 0.001). Ertapenem was the only agent to which there was no significant difference in rates of susceptibility between the *pks*-positive and *pks*-negative isolates (100% vs. 97.3%, *P* = 0.36).

### Microbiological and molecular factors associated with *pks*-positive isolates

Univariate analysis revealed that isolates of K1, K2, K20, and K62 capsular types, isolates from respiratory tract origin, and isolates less resistant were significantly associated with *pks*-carriage (Table 3). Multivariate analysis revealed that isolates of K1 [Odds ratio (OR) 1112.7, 95% confidence interval (95% CI) 103.535–11957], K2 (OR 187.456, 95% CI 19.813–1773.6,), K20 (OR 425.418, 95% CI 38.925–4649.5), and K62 (OR 508.24, 95% CI 42.06–6141.5) types (all *P* < 0.001), and being resistant to fewer than 1 or none of the antimicrobial agents tested (OR 18.281, CI 3.288–101.658, *P* = 0.001) remained independent factors strongly associated with *pks*-positivity.

### Clonality of *pks*-positive isolates and that of K1 and K2 isolates

**Figure 1:**
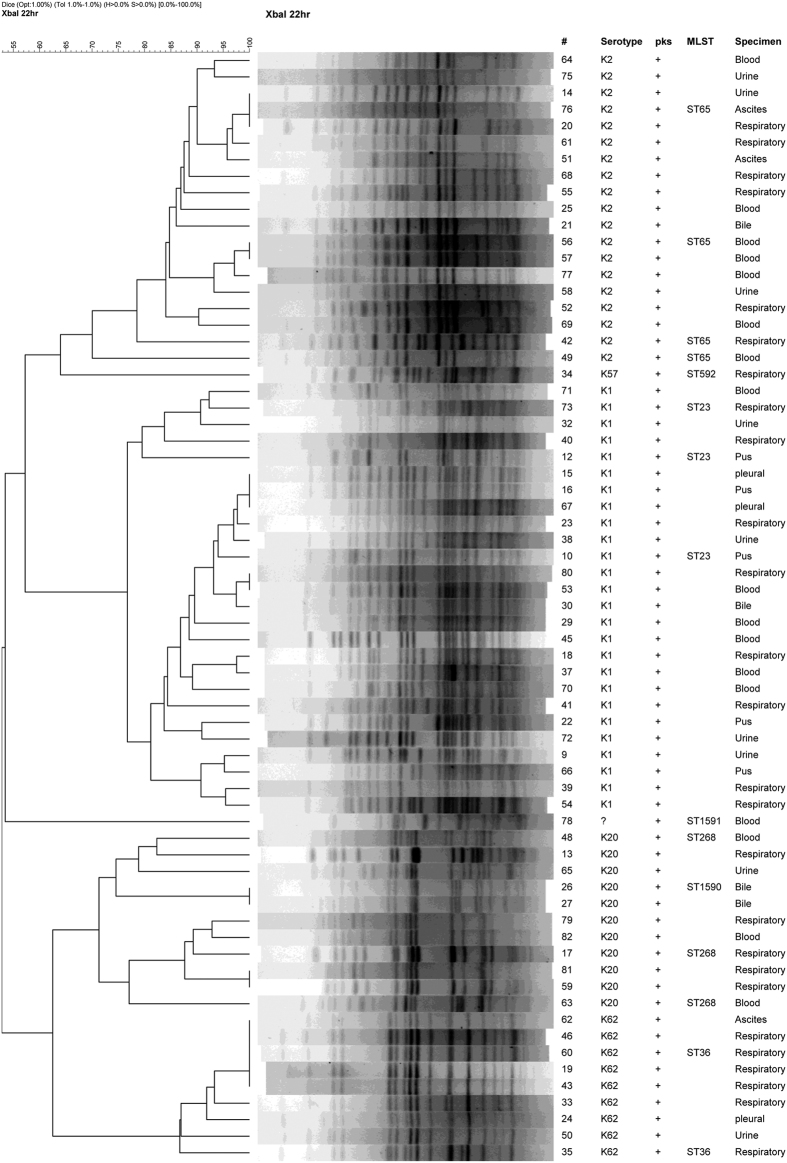
Dendrogram of the *pks*-positive *Klebsiella pneumoniae* clinical isolates. ^#^Isolate number; MLST, Multilocus sequence typing; ST, sequence type;?, capsular type unidentified.

Based on the pulsed-field gel electrophoresis (PFGE) pattern, the *pks*-positive isolates fall into 4 major clusters, with K1, K2, K20, and K62 isolates each belonging to its own distinct cluster (Fig. 1). Within the K1 isolates, two sub-clusters sharing >=80% similarity were seen but both sub-clusters belonged to sequence type (ST) 23. All but 2 of the *pks*-positive K2 isolates shared >=84% similarity in PFGE patterns and all were ST65 (including the 2 having <80% similarity in PFGE pattern). The K20 isolates were slightly more diverse in PFGE pattern, sharing 70% similarity, but had ST268 and a previously unreported new sequence type, ST1590, which is a single locus variant (SLV) of ST268. The *pks*-positive K62 (ST36) isolates were highly clonal with >=80% similarity in PFGE pattern including several isolates whose patterns were indistinguishable.

**Figure 2:**
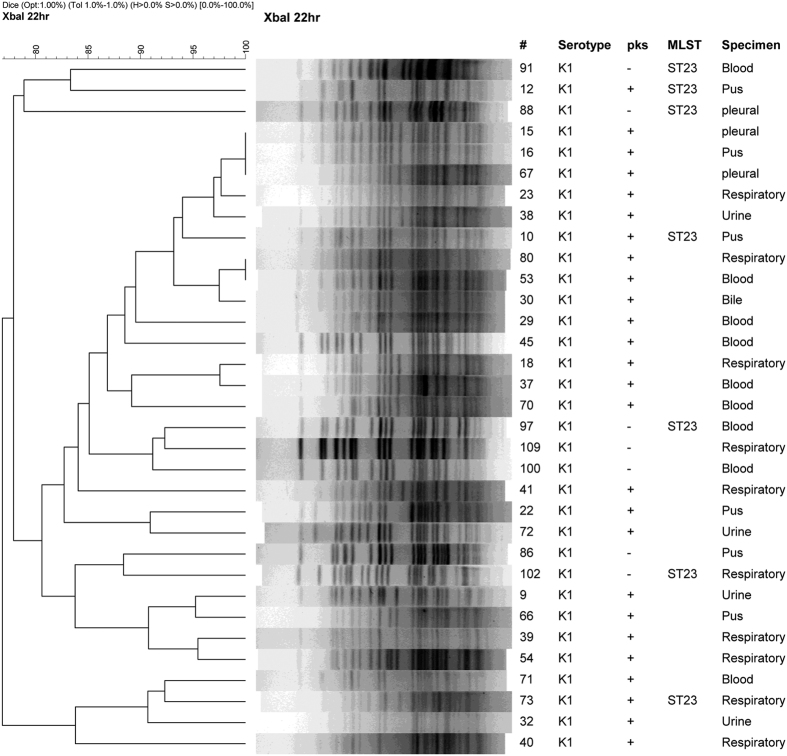
Dendrogram of *pks*-positive and *pks*-negative K1 capsular type *Klebsiella pneumoniae* clinical isolates. ^#^Isolate number; MLST, Multilocus sequence typing; ST, sequence type.

**Figure 3:**
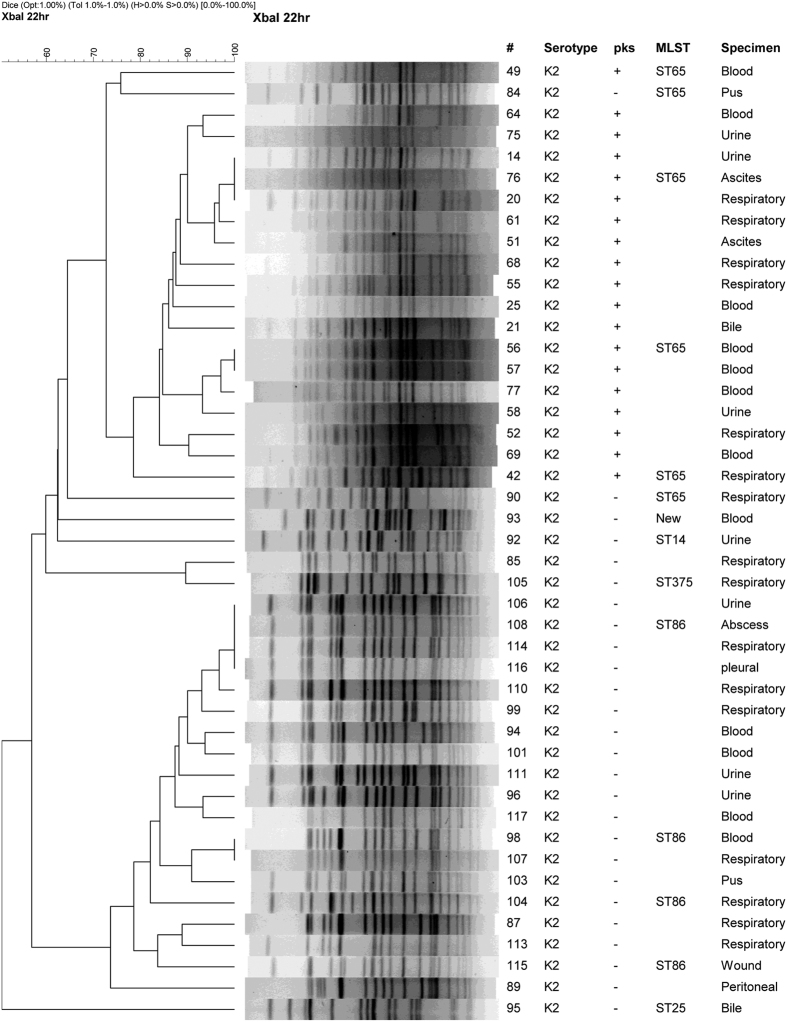
Dendrogram of *pks*-positive and *pks*-negative K2 capsular type *Klebsiella pneumoniae* clinical isolates. Sequence type (ST) 65 and ST86 differ by 5 out of the 7 alleles in the MLST (multilocus sequence typing) profile. The STs of the remaining *pks*-negative isolates are also distinct from ST65 and ST86. The new ST is a single locus variant of ST110.

Because K1 and K2 capsular types comprised the largest proportions of *pks*-positive isolates, and are independent factors associated with *pks*-positivity, we also investigated the clonality of all K1 and K2 isolates regardless of their *pks* status. PFGE clustering for isolates belonging to each of the two capsular types were performed (Figs 2 and 3). For K1 isolates, the *pks*-negative isolates are scattered in-between the *pks*-positive isolates in the tree. MLST analysis suggests that the *pks*-negative K1 isolates, like the *pks*-positive ones, are also all ST23 (Fig. 2). For K2 isolates, a distinct clade having <65% similarity to the rest of the K2 isolates were found to contain all of the *pks*-positive strains. The ST of the predominant *pks*-negative K2 isolates is ST86, which is distinct from the ST65 *pks*-positive K2 isolates. These 2 STs differ by 5 out of the 7 alleles in the MLST profile. The ST of the remaining *pks*-negative K2 isolates also differed from ST65 and ST86, and included ST14, ST25, ST375, and a new ST that is a SLV of ST110 (Fig. 3)

## Discussion

The *pks* gene cluster encodes enzymes that are responsible for the synthesis of colibactin, a genotoxin that has been shown to induce host DNA damage, thus may contribute to other disease entities and increased virulence in *E. coli* and *K. pneumoniae*^27^,^30^. However, data on the prevalence and microbiological factors associated with *pks*-positive *K. pneumoniae* isolates are limited. The present study showed that the prevalence of the *pks* colibactin gene cluster among clinical *K. pneumoniae* isolates from multiple hospitals in different regions of Taiwan was 16.7%. Our result corroborated the finding of high *pks* prevalence among *K. pneumoniae* by Lai *et al*.^28^, who studied isolates from a single hospital in Taiwan. Since the bacterial strains in our study were from 2012 and the study of Lai *et al*. used isolates from 2002, our results indicated persistence of the *pks* gene cluster in the clinical *K. pneumoniae* isolates in Taiwan.

While originally identified as a genotoxin targeting the host cells, the role of colibactin in the aspect of an emerging virulence pathogen lineage is just beginning to unfold. In *E. coli,* it was reported that the presence of the *pks* genes is strongly associated with bacteremia isolates, such as *E. coli* group B2^31^. Recently, it was shown that mice infected with colibactin-producing *E. coli* also had significantly lower survival rate than those infected with the isogenic colibactin-negative mutant^24^. It has been further demonstrated in a neonatal sepsis rat model, that colibactin contributes to the virulence of *pks*-positive *E. coli* in influencing its ability to colonize the gut of the neonate and to cause lethal invasive disease^32^. Our study revealed that *K. pneumoniae* isolates of K1, K2, K20, and K62 capsular types are independent factors strongly associated with *pks* carriage. K1, K2, K20 types have been associated with *Klebsiella* liver abscess (KLA) invasive disease and community-onset pneumonia, especially K1 and K2 isolates^3^,^16^,^17^. The *pks*-positive isolates in the present study were detected in different specimen types including blood, respiratory, urine, and abscess plus other specimen origins. Therefore, the *pks* gene cluster in *K. pneumoniae* may also contribute to invasive infections in different anatomical sites.

The virulence of the *pks*-positive *K. pneumoniae* isolates is further supported by our finding that 95.5% of them were also positive for *rmpA, ybt*, and *iutA*, the genes involved in hypermucoviscosity, yersiniabactin, and aerobactin production, respectively^7^,^18^,^19^,^20^,^21^,^22^. These virulence genes have been detected in large plasmid pLVPK homologs or genomic islands associated with hypervirulent *K. pneumoniae* clinical isolates^27^,^33^. Since nearly all of the genotoxic *K. pneumoniae* clinical isolates were positive for all three virulence factors (*rmpA, ybtA*, and *iutA*), it is plausible that the emerging *pks* genotoxic trait is associated with increased hypervirulent *K. pneumoniae* strains.

In *K. pneumoniae*, K1 and K2 strains have been regarded as the most virulent among its 79 capsular types^34^. In a recent study of 69 *K. pneumoniae* strains (including 32 K1 and 5 K2 type) from different regions of the world, genomic island variants carrying both *pks* colibactin and yersiniabactin siderophore-producing modules were found in nearly all hypervirulent K1 strains^27^. The genomic islands with both *pks* colibactin and yersiniabactin gene modules have also been identified in K2 strains but at much lower frequency^27^,^35^. It has been reported that the hypervirulent K1 strains mostly belong to ST23 or its SLV, whereas the hypervirulent K2 strains are more genetically diverse with different STs (ST65, ST86, and others)^10^,^27^,^36^,^37^. The present study also found that the K1:ST23 strains (33 isolates) were clonal regardless of their *pks* status based on PFGE. In contrast, the *pks*-positive (19 isolates) and *pks*-negative (26 isolates) K2 strains were segregated into two major clades, ST65 and ST86, respectively. The remaining *pks*-negative K2 isolates had different PFGE patterns and STs, including ST14, ST25, ST375, and a new ST that is a SLV of ST110. Our results suggest that the *pks*-positive lineage may have expanded locally among *K. pneumoniae* in Taiwan, especially a K2:ST65 clone, perhaps after acquisition of *pks* and associated genomic island modules that confer increased virulence or environmental fitness.

Of interest also was the *pks*-positive K62:ST36 isolates. These isolates came from 9 hospitals and are either indistinguishable or highly similar by PFGE. Reports on K62 capsular type *K. pneumoniae* are scarce. In a study of 592 *K. pneumoniae* isolates colonizing the intestinal tract of healthy Chinese and overseas Chinese adults in eight Asian countries, only 6 (1%) K62 were found^38^. The higher overall prevalence of K62 (25, 6.3%) in our 400 randomly selected clinical *K. pneumoniae* isolates might be due to differences in the study population. In addition, K62 comprised 13.4% of the *pks*-positive isolates in the present study. Therefore, more studies are needed to understand the epidemiology this capsular type in different disease entities in Taiwan.

We also found that isolates carrying the *pks* colibactin gene cluster are highly associated with low antimicrobial resistance. This is likely due in part to the fact that most of these isolates belong to K1 and K2 capsular types, as isolates of these capsular types recovered from KLA cases are usually less resistant to other antimicrobial agents^3^,^16^. However, multidrug-resistant hypervirulent *K. pneumoniae* isolates with extended spectrum β-lactamase (ESBL) or KPC carbapenemase have also been detected in different parts of the world^39^,^40^,^41^. The emergence of multidrug-resistance combined with genotoxicity in hypervirulent *K. pneumoniae* strains is worrisome. Careful monitoring of isolates with genotoxic colibactin *pks* gene cluster for acquired antimicrobial resistance is warranted.

In conclusion, the prevalence of *pks* colibactin gene cluster is high among clinical *K. pneumoniae* isolates in Taiwan. The finding of *pks*-positive hypervirulent isolates in different specimen types raised questions on the contribution of the genotoxic *K. pneumoniae* strains to the development of different disease entities. Since the isolates used in the present study were recovered from inpatients and outpatients from hospitals, thus may comprise more strains with resistance phenotypes, it is possible that the prevalence of *pks* colibactin gene cluster among *K. pneumoniae* strains in the general population in Taiwan may be even higher. Whether the genotoxic *K. pneumoniae* is associated with different disease entities in the locality requires extensive investigations. Further studies to determine the presence of *pks* gene bearing bacterial populations in the flora of community residents may also be warranted.

## Methods

### Isolates

*Klebsiella pneumoniae* isolates used in the present study were selected from the Taiwan Surveillance of Antimicrobial Resistance (TSAR) program conducted at the National Health Research Institutes (NHRI). The collection process for TSAR has been described previously and the 2012 isolates were collected from 27 hospitals located in different regions of Taiwan^29^. All isolates were stored at −80 °C at NHRI for subsequent testing. The species identification was confirmed using a combination of Triple Sugar Iron, indole test, and Vitek II GN card (bioMérieux, Marcy l’Etoile, France).

For determination of *pks* prevalence in the contemporary clinical *K. pneumoniae* isolate pool, the 400 isolates were randomly selected from the 831 *K. pneumoniae* isolates in the 2012 TSAR collection to include 100 each of 4 specimen categories: blood, respiratory, urine, and others. The others category included a mixture of isolates with a specimen source of ascites, abscess, bile, pleural, pus, tissue, wound, and miscellaneous sources.

### Antimicrobial susceptibility testing

Antimicrobial susceptibility testing was performed using broth microdilution method following the guidelines of the Clinical and Laboratory Standards Institute (CLSI) and the results were interpreted based on the CLSI breakpoints^42^.

### Detection of the *pks* gene cluster and hypervirulence genes in clinical *K. pneumoniae* isolates

The presence of the *pks* colibactin genes among 400 clinical *K. pneumoniae* isolates was determined by PCR using published primers^23^. Genomic DNA of each isolate was prepared by picking 3 to 5 colonies lightly from fresh overnight culture plate in 150 μl AE buffer [50 mM sodium acetate (pH 5.2) and 10 mM EDTA (pH 8.0)]. The suspension was heated at 95 °C for 15 min then centrifuged at 1000 g for 10 min to remove cellular debris, after which 100 μl of the supernatant was transferred to a new vial. The DNA preparation was stored at −20 °C and used as template for subsequent amplifications. Primer sets for the 3′ and 5′ regions of the *pks* colibactin gene cluster and its two internal loci were used for PCR following previously published protocols^28^. *K. pneumoniae* 1084 and NTUH K2044 were used as *pks*-positive and *pks*-negative controls, respectively.

To investigate the association of *pks* and hypervirulence, the presence of three hypervirulence genes, *iucA* (aerobactin), *rmpA* (hypermucositity), and *ybtA* (yersiniabactin) were determined by PCR following previously published protocols^43^,^44^. The primers used in the present study for *pks* and hypervirulence detection are listed in Supplemental Table 1 (Table S1).

### Determination of capsular types

All 400 isolates were first subject to capsular typing for K1, K2, K5, K20, K54, K57 using primers and protocols previously published^45^. For *pks*-positive isolates that were negative for K1, K2, K5, K20, K54, and K57, their capsular types were determined by PCR followed by sequencing of the *wzi* gene using previously published protocols^46^. Because 9 of the *pks*-positive isolates were K62, the *pks*-negative isolates were then also checked for K62 capsular type^47^. The primers are listed in Supplemental Table 1 (Table S1).

### Pulsed field gel electrophoresis (PFGE)

All *pks*-positive isolates and all K1 & K2 isolates (regardless of *pks* status) were subject to PFGE to determine strain relatedness. Plug preparation, restriction enzyme digestion, and electrophoresis conditions were performed as previously described^48^. Briefly, genomic DNAs of isolates were prepared and digested with 50 U *Xba*I (NEB, New England Biolabs Inc., Ipswich, MA, USA) at 37 °C for 16 to 18 h. The enzyme-digested DNA fragments were separated in 0.5 × Tris-Borate-EDTA buffer (pH 7.5) at 14 °C for 22 h with a voltage of 6 V/cm at a fixed angle of 120° and pulse times ranging from 2.16 to 54.17 s by CHEF MAPPER (Bio-Rad Laboratories, Richmond, CA). *Salmonella Choleraesuis Brenderup* H9812 (ATCC BAA664) was used as standard for DNA patterns normalization. PFGE patterns were analyzed using BioNumerics software (Applied Maths NV, Sint-Martens-Latem, Belgium).

### Multilocus sequence typing (MLST)

MLST was performed on isolates chosen from PFGE clusters and included each capsular type. The MLST procedure followed previously published protocols^49^ and information from the Institut Pasteur MLST website (http://www.pasteur.fr/mlst). Sequence types (STs) were assigned using the *Klebsiella* MLST database. New ST information was deposited at the Institut Pasteur MLST web site.

### Ethics statement

The bacterial isolates were recovered from clinical samples taken as part of standard care. The TSAR project was approved by the Research Ethics Committee of National Health Research Institutes (EC1010602-E). The study methods were performed in accordance with the relevant guidelines and regulations.

### Data analysis

Susceptibility interpretation analysis was made using the WHONET software^50^. Significance of differences in frequencies and proportions was tested by the *χ*^2^ test or Fisher’s exact test (if the number was less than 10). Predictor variables associated with *pks*-carriage investigated included capsular type (K1, K2, K20, K57, K62 vs. other), age (<=18 y.o.; 19–64 y.o., >=65 y.o.), specimen source (blood, respiratory, urine, and others), and antimicrobial susceptibility (being resistant to one or none of the antimicrobial agents tested). Multivariable logistic regression analysis was performed to assess the relationship between predictor variables among *pks*-positive and *pks*-negative isolates. All analyses were performed with the Statistical Package for the Social Sciences version 18.0 (SPSS, Chicago, IL, USA). A *P* < 0.05 was considered to be statistically significant.

## Additional Information

**How to cite this article:** Chen, Y.-T. *et al*. Prevalence and characteristics of *pks* genotoxin gene cluster-positive clinical *Klebsiella pneumoniae* isolates in Taiwan. *Sci. Rep.* **7**, 43120; doi: 10.1038/srep43120 (2017).

**Publisher's note:** Springer Nature remains neutral with regard to jurisdictional claims in published maps and institutional affiliations.

## Supplementary Material

Supplementary Information

## Figures and Tables

**Table 1:** Prevalence and capsular type distribution of *pks* colibactin gene cluster in clinical *Klebsiella pneumoniae* isolates stratified by specimen type.

Capsular type^a^ (n)	No. of isolates in each specimen group (no. of isolates *pks* positive)				Capsular type prevalence^b^	*pks* prevalence^c^
	Blood (n = 100)	Respiratory (n = 100)	Urine (n = 100)	Others (n = 100)		
K1 (33)	9 (6)	10 (8)	4 (4)	10 (8)	8.3 (33/400)	78.8 (26/33)
K2 (45)	12 (7)	16 (6)	7 (3)	10 (3)	11.3 (45/400)	42.2 (19/45)
K5 (12)	4 (0)	6 (0)	0	2 (0)	3.0 (12/400)	0 (0/12)
K20 (23)	4 (3)	10 (5)	4 (1)	5 (2)	5.8 (23/400)	47.8 (11/23)
K54 (20)	5 (0)	2 (0)	8 (0)	5 (0)	5.0 (20/400)	0 (0/20)
K57 (8)	1 (0)	6 (1)	0	1 (0)	2.0 (8/400)	12.5 (1/8)
K62 (25)	3 (0)	7 (6)	6 (1)	9 (2)	6.3 (25/400)	36.0 (9/25)
Unknown (234)^a^	64 (1)	58 (0)	43 (0)	68 (0)		0.4 (1/234)

^a^In the 234 non-K1, K2, K5, K20, K54, K57, K62 isolates, capsular type analysis was carried out on the 1 *pks*-positive isolate but its capsular type could not be identified. ^b^Number of isolates with the capsular type/400 isolates studied. ^c^Number of isolates positive for *pks*/number of isolates within each capsular type.

**Table 2:** Antimicrobial susceptibility of *pks*-positive and *pks*-negative *Klebsiella pneumoniae* isolates.

Antimicrobial agent^a^	N (%) of *pks*-positive isolates (n = 67)		N (%) of *pks*-negative isolates (n = 333)		*p*
	Resistant^b^	Susceptible	Resistant^b^	Susceptible	
Amikacin	0	67 (100.0)	25 (7.5)	306 (91.9)	0.013
Aztreonam	0	67 (100.0)	68 (20.4)	260 (78.1)	<0.001
Ceftazidime	0	67 (100.0)	68 (20.4)	245 (73.6)	<0.001
Cefazolin	4 (6.0)	63 (94.0)	109 (32.7)	221 (66.4)	<0.001
Ciprofloxacin	0	67 (100.0)	92 (27.6)	232 (69.7)	<0.001
Cefepime	0	66 (98.5)	40 (12.0)	283 (85.0)	0.005
Cefoxitin	3 (4.5)	63 (94.0)	82 (24.6)	236 (70.9)	<0.001
Cefuroxime	5 (7.5)	62 (92.5)	104 (31.2)	221 (66.4)	<0.001
Cefotaxime	1 (1.5)	66 (98.5)	58 (17.4)	259 (77.8)	<0.001
Ertapenem	0	67 (100.0)	8 (2.4)	324 (97.3)	0.36
Gentamicin	0	67 (100.0)	84 (25.2)	239 (71.8)	<0.001
Piperacillin/Tazobactam	0	67 (100.0)	61 (18.3)	256 (76.9)	<0.001
Ticarcillin/Clavulanate	2 (3.0)	62 (92. 5)	100 (30.0)	215 (64.6)	<0.001
Trimethoprim/Sulfamethoxazole	1 (1.5)	66 (98.5)	120 (36.0)	213 (64.0)	<0.001

^a^Ampicillin was also tested but not included in analysis because isolates of *K. pneumoniae* have intrinsic resistance to this agent. ^b^Resistant data do not include intermediate results.

**Table 3:** Factors associated with carriage of *pks* gene cluster in *Klebsiella pneumoniae* clinical isolates.

Variable (n)	No. (%) of isolates		*P*^a^	OR^b^	95%CI^b^	*P*^b^
	*pks*-Positive	*pks*-Negative				
Total (400)	67	333				
**Age**			0.719			
< = 15 y.o. (18)	2 (3.0)	16 (4.8)	0.749			
16–64 y.o. (163)	26 (38.8)	137 (41.1)	0.723			
> = 65 y.o. (219)	39 (58.2)	180 (54.1)	0.533			
**Specimen source**			**0.014**			
Blood (100)	17 (25.4)	83 (24.9)	0.938	2.525	0.644–9.902	0.184
Respiratory (100)	26 (38.8)	74 (22.2)	0.004	1.501	0.387–5.828	0.557
Urine (100)	9 (13.4)	91 (27.3)	0.017		Reference	
Abscess & Others (100)	15 (22.4)	85 (25.5)	0.588	0.809	0.203–3.222	0.763
**Capsular type**			**<0.001**			
K1 (33)	26 (38.8)	7 (2.1)	<0.001	1112.7	103.535–11957	<0.001
K2 (45)	19 (28.4)	26 (7.8)	<0.001	187.456	19.813–1773.6	<0.001
K20 (23)	11 (16.4)	12 (3.6)	<0.001	425.418	38.925–4649.5	<0.001
K57 (8)	1 (1.5)	7 (2.1)	1	25.798	1.16–573.73	0.04
K62 (25)	9 (13.4)	16 (4.8)	0.022	508.24	42.06–6141.5	<0.001
Non-K1, K2, K20, K57, K62 (266)	1 (1.5)	265 (79.6)	<0.001		Reference	
**Antimicrobial resistance**			**<0.001**			
Resistant to <= 1 agent (272)	65 (97.0)	207 (62.2)		18.281	3.288–101.658	0.001
Resistant to >= 2 agents (128)	2 (3.0)	126 (37.8)			Reference	

^a^*P* value by univariate analysis. Only variables having statistical significance by univariate analysis were subject to multivariate analysis. ^b^*P* value by multivariate analysis; OR, odds ratio; CI, confidence interval.
