# Supplementary material for: Prevalence and characteristics of pks genotoxin gene cluster-positive clinical Klebsiella pneumoniae isolates in Taiwan
Source: Sci Rep. 2017 Feb 24;7:43120. doi: 10.1038/srep43120 (PMC5324043; doi:10.1038/srep43120)
Supplement: Supplementary Information [file srep43120-s1.pdf]

Prevalence and characteristics of *pks* genotoxin gene cluster-positive clinical *Klebsiella pneumoniae* isolates in Taiwan

Ying-Tsong Chen, Yi-Chyi Lai, Mei-Chen Tan, Li-Yun Hsieh, Jann-Tay Wang, Yih-Ru Shiau, Hui-Ying Wang, Ann-Chi Lin, Jui-Fen Lai, I-Wen Huang, Tsai-Ling Lauderdale

Table S1. PCR primers used for studying the *pks* status, hypervirulence, and capsular type of *Klebsiella pneumoniae* isolates.

Target	Primers	Nucleotide sequence (5’- 3’)	Reference
pks(*clbB*)	clbBF	GATTTGGATACTGGCGATAACCG	Nougayrede 2006
	clbBR	CCATTTCCCGTTTGAGCACAC	
pks(*clbN*)	clbNF	GTTTTGCTCGCCAGATAGTCATTC	Nougayrede 2006
	clbNR	CAGTTCGGGTATGTGTGGAAGG	
pks(*clbA*)	clbAF	CTAGATTATCCGTGGCGATTC	Nougayrede 2006
	clbAR	CAGATACACAGATACCATTCA	
pks(*clbQ*)	clbQF	CTTGTATAGTTACACAACTATTTC	Nougayrede 2006
	clbQR	TTATCCTGTTAGCTTTCGTTC	
Aerobactin	iutAF	ACCTGGGTTATCGAAAACGC	Tang 2010
	iutAR	GATGTCATAGCCTGATTGC	
Yersiniabactin	ybtAF	ATGACGGAGTCACCGCAAAC	Hsieh 2008
	ybtAR	TTACATCACGCGTTTAAAGG	
rmpA	rmpAF	ACGACTTTCAAGAGAAATGA	Tang 2010
	rmpAR	CATAGATGTCATAATCACAC	
K1 capsular type	MagAF1	GTAGGTATTGCAAGCCATGC	Fang 2007
	MagAR1	GCCCAGGTTAATGAATCCGT	
K2 capsular type	K2wzy-F1	GGAGCCATTTGAATTCGGTG	Fang 2007
	K2wzy-R1	TCC CTAGCACTGGCTTAAGT	
K5 capsular type	K5wzxF360	TGGTAGTGATGCTCGCGA	Turton 2008
	K5wzxR639	CCTGAACCCACCCCAATC	

K20 capsular type	wzyK20F	CGGTGCTACAGTGCATCATT	Fang 2007
	wzyK20R	GTTATACGATGCTCAGTCGC	
K54 capsular type	wzxK54F	CATTAGCTCAGTGGTTGGCT	Fang 2007
	wzxK54R	GCTTGACAAACACCATAGCAG	
K57 capsular type	wzyK57F	CTCAGGGCTAGAAGTGTCAT	Fang 2007
	wzyK57R	CACTAACCCAGAAAGTCGAG	
wzi	wzi_F2	GTGCCGCGAGCGCTTTCTATCTTGGTATTCC	Brisse 2013
	wzi_rev	GAGAGCCACTGGTTCCAGAAYTTSACCGC	

Y: C or T
S: C or G
